# Effects of coronavirus (COVID-19) pandemic on orthopedic residency program in the seventh largest city of the world: Recommendations from a resource-constrained setting

**DOI:** 10.1016/j.amsu.2020.06.026

**Published:** 2020-06-25

**Authors:** Marij Zahid, Arif Ali, Naveed Juman Baloch, Shahryar Noordin

**Affiliations:** Orthopedic Surgery, Aga Khan University Hospital, Karachi, Pakistan

**Keywords:** Coronavirus, Quarantine, Orthopedic surgeons, Pandemics, Medical education

## Abstract

The coronavirus (COVID-19) pandemic has hit the entire world hard. Since its inception from Wuhan China the whole world is affected now. Health care facilities and workers are overwhelmed and the situation is changing on daily basis. With the changes in the dynamics of the hospitals, residency and fellowships training programs have also suffered undoubtedly. Due to decreased elective cases and outpatient clinics surgical training gets compromised, however on the other side this physical distancing and isolation have proven to be effective measures in controlling the disease. In this article we share our experience of effect of COVID-19 pandemic on our orthopedic residency program and how we coped along with it. We also discussed some way forwards in the article.

## 1 Introduction

Since the inception of novel coronavirus in Wuhan, China, the global situation has changed dramatically in every sector of life. The changes are dynamic and constantly evolving, even more so as our country has not yet reached the peak, as we were quick to learn and implement social distancing from the world. Furthermore, our government has been following the process of screen, test and quarantine for confirmed cases which has resulted in containing the disease. In the beginning of March 2020, Pakistan sealed its borders with China, Iran and Afghanistan and international arrivals through flights were also curtailed as our airports were closed. Our hospital diagnosed the first COVID-19 PCR positive case in the country on February 26 who was a student returned from Iran [1]. Our section of Orthopedic Surgery was the first in our tertiary care hospital to cut down outpatient clinic numbers by restricting practice to only “must be seen” follow-up patients especially who have underwent surgery in prior weeks and so on, even before our University hospital published their guidelines. As of morning of May 17, total corona virus cases in Pakistan are 40,151 with 873 mortalities [1]. The city of Karachi, where our tertiary care hospital is located is in lock down since March 23, which as per the last government announcement was switched to a smart lockdown (re-opening of some semi-essential sectors) on May 11.

The two vital practices of “social distancing” and “quarantine” have been key to control the situation both in our community and hospital setup [2]. Health care workers being at the front line are more likely to contract the virus due to the current pandemic; yet it is also important to maintain a healthy resident workforce to sustain the ongoing patient care. As orthopedic surgeons are not the usual front line health care providers, adapting to these protective practices in our day to day inpatient and outpatient clinical work faced us with several challenges.

Various practices have been adopted by the hospitals worldwide to fight this issue which included suspension of elective surgeries like knee arthroplasty, minimizing outpatient physical clinics to suture and cast removal patients, using telemedicine to see new patients, suspension of physical academic meetings and tele-academic sessions. These practices on one hand minimize the risk of transmission of virus, tend to conserve personal protective equipment but on the other hand have a dilutional academic effect on the residency programs more so in terms of real time patient assessment and surgical hands on skills [3]. In the few past months, the surgical residency program has suffered a lot worldwide in terms of suspension of elective cases as a sequelae of succumbing to a lower patient load [4]. Despite the lower patient surgical hands on for residents however, this pandemic has a brighter side in that it has opened multiple positive avenues and learning opportunities for residents that were never explored before. It is still unpredictable when this pandemic will be under control and the situation slowly normalizes. Will there be a second peak and how badly would it hurt? Everyone is currently living in such ambiguity. We in this article share our experience of orthopedic residency program in an urban tertiary care center and delineate our division of work force during this pandemic and propose few recommendations.

## 2 Resident workforce distribution

We have a total of 18 residents in Orthopedic Surgery out of whom 16 are currently enrolled in orthopedic residency program with 2 rotating residents one each from General Surgery and Emergency Medicine programs. Prior to the COVID-19 pandemic, our call schedule was one in four. Once the pandemic struck, as per guidelines from university's department of postgraduate medical education, our resident workforce was divided in two teams from March 25, 2020 onwards: team A and team B which are working on alternate weeks. We actually put our residents on a one week on and one week off work schedule to limit the overall exposure. Both of the teams were further subdivided into team A1 and A2 and B1 and B2 respectively. Each group of teams comprises of 4 residents, leaving two residents as back up in a hospital wise pool of residents utilized for replacing the residents who are quarantined due to COVID-19 exposure or by other specialties where required. This practically worked out to every alternate day call for one week followed by off for the next week. The on call residents are expected to work in theatres, manage admitted patients and review orthopedic consultations in emergency room and wards. Residents were not expected to attend outpatient physical or tele-clinics which were manned by attending and nursing staff. This strategy provided adequate daily workforce and safety from contacting the virus. Luckily none of our orthopedic resident tested positive although 6 got exposed and tested negative.

## 3 Academic meetings

Prior to the COVID pandemic, we had daily academic activities which included a faculty lecture, department residency committee meeting, research cell meeting, morbidity and mortality meeting on each of four Monday mornings, Radiology review meetings on Tuesdays and Thursdays, Resident Review of core curriculum topics on Wednesdays and a Multiple choice question session/Physiotherapy research retreat biweekly on Fridays. On weekend Saturdays thrice a month we had sessions including two workshop and one journal club. All these sessions were conducted between 8 a.m. and 9:30 a.m. and reporting time for residents was 7am. In that 1 h between 7 a.m. and 8 a.m. residents used to do an indication meeting for first 30 min supervised by chief resident; discussing important cases admitted by on call team. Last 30 min were utilized to do ward round and see patients they are responsible for. These were all sessions where there were physical congregations. Post COVID pandemic, all these academic meetings (except workshops and morbidity and mortality meeting) have been switched to virtual conferences via Zoom or Microsoft teams. These academic activities are usually lead by team of residents not involved in service in that week. Due to the low patient workload, we had more participation both from the residents and faculty in these meetings as compared to pre-COVID days. Initially there were some glitches and hiccups in the use of new teaching modality as all participants had to get acquainted with the technology. As we progressed along the learning curve, there came the ease and freedom of expression, with high level academic discussions. The junior residents found this modality particularly fascinating, as distant learning decreased their performance anxiety and improved their output and learning.

## 4 Outpatient clinics

Once the COVID pandemic came in our attending decreased the number of physical clinics per week and were only seeing follow up patients that were necessary to see, such as those required postop wound evaluations, suture and cast removals etc. Prior to the COVID pandemic, we conducted tele-clinics for outstation patients, but after being hit by the pandemic we started virtual clinic consultations for local patients as the general public was scared and hesitant to come to the hospital. The patients being scheduled in the clinic were screened on the telephone prior to be given an appointment for any complaints and symptoms of COVID like recent fever, cough, generalized body aches, recent travelling history form high risk areas. Once these follow up patients reached the hospital, prior to seeing the consultant, they were again screened using the questionnaire. This has two-fold advantage of reducing the risk of contamination to the health care workers and exposure of the patients from hospital environment. Post-operative visits are limited until the opening of sutures. Immediate post-op wound assessment is done by the home-health care team in concert with the primary physician. In case of wound complications, patient is scheduled for an urgent follow up as per the surgeon's discretion. Cutting short the clinic load resulted in decreasing patient volumes by almost 70%. Since the hospital COVID policy changed to minimal personnel in clinic, residents were not supposed to attend clinics. This compromises their major chance of history taking, physical examination and assessment, hence leading to a significant deficiency in completing their clinic based assessment and outpatient procedural skills like adjustment of Ilizarov fixators, out-patient clinic biopsies and removal of k-wires etc. To overcome this, we are in the process of designing and planning to commence residents' virtual clinic portfolio.

## 5 Surgical procedures and hands on

With the rising surge of COVID pandemic, the elective cases which are the major bulk of orthopedic surgery have been suspended and postponed for indefinite period. Our orthopedic facility has been operating emergency cases like fractures and infections or semi-urgent cases like tumors which cannot wait, for the past one month resulting in a significant decrease in total cases. We found 50% reduction in surgical volume in orthopedic surgery in the COVID era as compared to the pre-COVID era. We compared the cases done in six week before lockdown (1st February 2020 to 15th March 2020) with cases performed in six week duration after the lockdown (16th March 2020 to 30th April). The cut in total number of cases especially elective ones resulted in decrease in residents’ hands on experience.

## 6 Research activities and research ethical review committee decisions

Due to decreased workload, our residents got more time to work on their unfinished research projects. The University Research Office significantly reduced the turnaround time for processing research grants from 4 weeks to 7 days. Our Section applied for three grants during this period.

## 7 Recommendations and way forward

The significant learning for our program was that we embraced social distancing by restricting our elective outpatient and surgical work even before it was mandated by the hospital and our federal and provincial regulations. This has significantly reduced our case volumes and we have not yet reached the peak. Currently in the setting of low volumes, we have planned to increase the number and duration of indication meetings and pre-operative planning sessions. Residents have more time to spend on arthroscopy simulators in our Center for Innovation in Medical Education. Currently we are in the upsurge phase of the pandemic and different mathematical models have projected that we may see the peak in the next two months, depending upon which model is considered. If this pandemic turns into an endemic in our part of the world in the near future, then we should be able to accommodate for the time lost, otherwise strategies such as increasing the duration of the residency program may have to be considered. This approach would seems to be essential to make up for loss of resident hands on and clinical experience before they step into their surgical career, in order to ensure we graduate safe and appropriately trained surgeons from the program.

Despite of decreased clinical work, our hospital has not resorted to layoffs and furloughs. 77% bedside care staff will not take any pay cuts. Senior level faculty and staff including management will have pay cuts that have been announced for three months with review thereafter. This has allowed us to manage our workforce and set policies in place to ensure we hit the road running when the full force of the pandemic hits us. We have already developed a COVID screening zone for patients which is cordoned off from the main hospital. For COVID patients we have isolated a building comprising of three floors to house these patients in isolation with appropriate special cares and ventilator support. We have 20 operating suites, 3 of which cater to day care surgery. The day care surgery operating suites were closed down and to date are not operational. Three operating room theaters were changed to negative pressure rooms to cater to corona positive cases. To date there is no data to show that viral particles are present in bone and bone marrow. Nevertheless in cases where aerosols are anticipated we use full PPE including N95, face shields and water impermeable gowns in addition to double gloving. Our policies and guidelines for COVID related issues are constantly being updated and the coming weeks will be crucial to see if the lessons learned internationally will help us get through this pandemic safely.

## Provenance and peer review

Not commissioned, externally peer reviewed.
